# Supplementary material for: Pesticide use safety practices and associated factors among farmers in Fogera district wetland areas, south Gondar zone, Northwest Ethiopia
Source: PLoS One. 2023 Jan 10;18(1):e0280185. doi: 10.1371/journal.pone.0280185 (PMC9831305; doi:10.1371/journal.pone.0280185)
Supplement: S2 Data — (DOCX) [file pone.0280185.s002.docx]

1. Structured questionnaire for face to face interview

Socio-demographic questions:

Code--------- living place: Urban---- Rural----- house number-----------

| s.no | List of questions | Key answers | remark |
| --- | --- | --- | --- |
| 101 | Sex | 1. Male 2. Female |  |
| 102 | Age | 1. < 18 2. 30-39  3. 40-49 4. 50-59  5. ≥60 |  |
| 103 | The head of household: | 1. Father 2. Mother 3. son |  |
| 104 | Religion | 1. Orthodox 2. Muslim  3. Catholic 4. Protestant  5. other |  |
| 105 | Educational level | 1. Unable to read and write  2. Can read and write  3. Primary school(1-8)  4.Secondschool(9-12) 5.Diploma and above |  |
| 106 | Marital status | 1. Single 2. Married  3. Divorced 4. Widowed |  |
| 107 | Family size | 1. Single 2. Two  3. Three 4. four  5. Fife and above |  |
| 108 | Years of Experience in pesticide spray | 1. 6month -5 years 2. 6- 10 years  3. 11-15 years 4. 16-20 years  5. > 20 years |  |
| 109 | Average Monthly Income | 1. 1000-1500 birr 2. 1501-2000 birr  3. 2001- 2500 birr 4. 2501-3000 birr  5. ከ3000 birr and above |  |
| 110 | Working hours per day on the farm | 1. As necessary 2. 8hours  3. greater than 8hours |  |
| 111 | Farm sizes (in hectare) | 1. <1.0  2. 1.0  3. >1.0 |  |
| 112 | Land tenure situation | 1. Landowners 2. Land holders |  |
| 113 | Trend of pesticide use in the past 5 years | 1. Increasing 2. Constant |  |

Knowledge related questions about pesticide:

| s.no | List of questions | Key answers | | Remark |
| --- | --- | --- | --- | --- |
| 201 | You know the names of pesticides you used | 1. No | 2. Yes |  |
| 202 | You know that pesticides affect human health | 1. No | 2. Yes |  |
| 203 | You know that pesticides affect livestock | 1. No | 2. Yes |  |
| 204 | You know that pesticides affect environment (water bodies) | 1. No | 2. Yes |  |
| 205 | You ever read pesticide labels | 1. No | 2. Yes |  |
| 206 | You know Crop-wise pesticide use | 1. No | 2. Yes |  |
| 207 | You have knowledge about “Guideline for safety application of pesticides” | 1. No | 2. Yes |  |
| 208 | You know contaminated water with pesticides make people sick | 1. No | 2. Yes |  |
| 209 | You know routes of pesticides can enter your body | 1. No | 2. Yes |  |
| 210 | If yes, what are they? | 1. Skin  2. Inhalation  3. Mouth  4. Eye | |  |
| 211 | You know, you will be exposed to pesticide if you entered the farm after a few hours of spraying (2–3 hours) | 1. No | 2. Yes |  |
| 212 | You have previous knowledge on pesticides use safety | 1.No | 2. Yes |  |
| 213 | You can read and understand toxic label present in the pesticide containers | 1.No | 2. Yes |  |
| 214 | You follow the instructions and recommended dose on the label | 1. No | 2. Yes |  |
| 215 | You have awareness about types of prohibited pesticides | 1. No | 2. Yes |  |
| 216 | You know effect of pesticide residuals exist | 1. No | 2. Yes |  |
| 217 | If yes on which part? | 1. Air 2. Soil 3. Water  4.Cereals/legumes 5.Vegetables | |  |
| 218 | You have taken about training on safe utilization of pesticides | 1. No | 2.Yes |  |
| 219 | You have received support from district agricultural office | 1. No | 2. Yes |  |
| 220 | You have taken place safety communication about pesticide with other farmers | 1.No | 2. Yes |  |

Attitude related questions

| s.no | List of questions | Key answers | | | | | remark |
| --- | --- | --- | --- | --- | --- | --- | --- |
| 301 | You fear pesticides affect your health | 1.Strongly disagree | 2.Disagree | 3. I don’t know | 4. Agree | 5.Strongly agree |  |
|  |  |  |  |  |  |  |  |
| 302 | You give attention to information written on pesticide packages | 1.Strongly disagree | 2.Disagree | 3. I don’t know | 4. Agree | 5.Strongly agree |  |
| 303 | You are interested to wear protective equipments | 1. Strongly disagree | 2. Disagree | 3. I don’t know | 4. Agree | 5.Strongly agree |  |
| 304 | You have positive feeling towards instructions about safe pesticide handling methods | 1. Strongly disagree | 2. Disagree | 3. I don’t know | 4. Agree | 5. Strongly agree |  |
| 305 | Perceiving that safe use of pesticides protect the environment | 1. Strongly disagree | 2. Disagree | 3. I don’t know | 4. Agree | 5. Strongly agree |  |
| 306 | Interested to buy safety equipments | 1. Strongly disagree | 2. Disagree | 3. I don’t know | 4. Agree | 5. Strongly agree |  |
| 307 | You are interested to wash hands after pesticide spraying | 1. Strongly disagree | 2. Disagree | 3. I don’t know | 4. Agree | 5. Strongly agree |  |
| 308 | You like to drink water during spraying pesticides | 1. Strongly disagree | 2. Disagree | 3. I don’t know | 4. Agree | 5. Strongly agree |  |
| 309 | You are interested to take shower after spraying pesticides | 1. Strongly disagree | 2. Disagree | 3. I don’t know | 4. Agree | 5. Strongly agree |  |
| 310 | You give attention to share ideas about safety pesticide use practices | 1. Strongly disagree | 2. Disagree | 3. I don’t know | 4. Agree | 5. Strongly agree |  |
| 311 | You are interested to change clothes you have used during spraying pesticide | 1. Strongly disagree | 2. Disagree | 3. I don’t know | 4. Agree | 5. Strongly agree |  |

Environmental factor related questions

| s.no | List of questions | Key answers | | remark |
| --- | --- | --- | --- | --- |
| 401 | You have care of wind direction while spraying pesticides | 1. No | 2. Yes |  |
| 402 | Where do you store the pesticides? | 1.In the bedroom  2.In the living room  3. In the kitchen  4. In a locked and separate place | |  |
| 403 | What is the length of time use store the pesticides? | 1. 6months  2.6-12 months  3.12-24 months  4.For unlimited length of time | |  |
| 404 | You dispose empty pesticide containers | 1. No | 2. Yes |  |
| 405 | If you say yes? how do you dispose it? | 1. Burning  2. Burring  3. Leave on farm land  4. Dispose in to rivers | |  |

| Practices related questions   \| s.no \| List of questions \| Key answers \| \| \| \| remark \| \| --- \| --- \| --- \| --- \| --- \| --- \| --- \| \| 501 \| You ever used pesticides previously \| 1. No \| \| 2. Yes \| \|  \| \| 502 \| You have currently use pesticides \| 1. No \| \| 2. Yes \|  \|  \| \| 503 \| What kind of pesticides you used? \|  \| \|  \| \|  \| \| 504 \| You have used mixed combination of pesticides \| 1.No \| \| 2. Yes \| \|  \| \| 505 \| If you say yes, which type of pesticides you mix? \|  \| \| \| \|  \| \| 506 \| Where do you mixing pesticide? \| 1. near a river canal/community water sources  2. In the field (farm)  3. At home \| \| \| \|  \| \| 507 \| How do you mix pesticides \| 1. With a stick, but bare hands  2. With bare hands  3. With hands and wearing gloves  4. With a stick and wearing gloves \| \| \| \|  \| \| 508 \| You have usually used a measuring cup or measuring tool to add the exact amount of pesticide mentioned on the label \| 1. No \| \| 2. Yes \| \|  \| \| 509 \| What type of devices used for mixing pesticides? \| 1. Knapsack \| 2. Bucket \|  \| \|  \| \| 510 \| How many times do you spray pesticides in a year? \| 1.2 \| 2. 3 \| 3. 4 and above \| \|  \| \| 511 \| Where do you have provision of PPE? \| 1.From government organization  2. NGOs  3. Buying from retailers 4. Not used \| \| \| \|  \| \| 512 \| You have applied of health and safety instructions \| 1. No \| \| 2. Yes \| \|  \| \| 513 \| You have used safe procedures regularly \| 1. No \| \| 2. Yes \| \|  \| \| 514 \| You have regularly used PPE \| 1. No \| \| 2. Yes \| \|  \| \| 514 \| If your answer yes, which items used? \| 1. Mask 2. Goggle  3. Glove 4. Boot  5. Long sleeved shirt  6. Long trousers 7. Coverall \| \| \| \|  \| |
| --- | --- | --- | --- | --- | --- | --- | --- | --- | --- | --- | --- | --- | --- | --- | --- | --- | --- | --- | --- | --- | --- | --- | --- | --- | --- | --- | --- | --- | --- | --- | --- | --- | --- | --- | --- | --- | --- | --- | --- | --- | --- | --- | --- | --- | --- | --- | --- | --- | --- | --- | --- | --- | --- | --- | --- | --- | --- | --- | --- | --- | --- | --- | --- | --- | --- | --- | --- | --- | --- | --- | --- | --- | --- | --- | --- | --- | --- | --- | --- | --- | --- | --- | --- | --- | --- | --- | --- | --- | --- | --- | --- | --- | --- | --- | --- | --- | --- | --- | --- | --- | --- | --- | --- | --- | --- | --- | --- | --- | --- | --- | --- | --- |
| \| 515 \| If say no, What are reasons for not using protective equipment \| 1. uncomfortable  2. Too expensive to buy  3. Time-consuming to use  4. Not available when needed  5. not necessary for each case \| \|  \| \| --- \| --- \| --- \| --- \| --- \| \| 516 \| You usually check the defect (inadequacy) of the PPE before dealing with pesticides \| 1. No \| 2. Yes \|  \| \| 517 \| What do you use/take/ during spraying? \| 1. Nothing  2. Chew chat  3. Drink alcohol  4. Smoke cigarette \| \|  \| \| 518 \| You change cloths after spraying pesticides \| 1. No \| 2. Yes \|  \| \| 519 \| You take shower after spraying pesticide \| 1. No \| 2. Yes \|  \| \| 520 \| If you say yes when? \| 1. Some times \| 2. Always \|  \| \| 521 \| You have symptoms after spraying \| 1. No \| 2. Yes \|  \| \| 522 \| If say yes what may be the most common symptoms feel? \| 1.Skin rash  2. Headache  3. Skin and eye burning  4. Cough  5. Change of mood \| \|  \| |

OBSERVATION CHECKLIST

Date of inspection---------------------- code-----------------

Education level: educated ---- uneducated ----

Work experience: 20 years and above ----- less than 5 years---

| Ser.no | Elements of checklists | Yes | No | Remark |
| --- | --- | --- | --- | --- |
|  | **Regarding pesticides package** |  |  |  |
| 1 | Container caps are tightly closed. |  |  |  |
| 2 | Labels of pesticides readable. |  |  |  |
| 3 | Date of expired mentioned. |  |  |  |
| 4 | Safety pictograms explained. |  |  |  |
| 5 | Type of pesticides with pests listed. |  |  |  |
| 6 | The rate of mixture described. |  |  |  |
| 7 | The amount of pesticide and hectare ratio is mentioned. |  |  |  |
|  | **Regarding mixing** |  |  |  |
| 1 | The mixture is applied based on the rate listed on the label. |  |  |  |
| 2 | The mixer used safety equipments mentioned on the label. |  |  |  |
| 3 | The area was Ventilated where chemicals are mixed. |  |  |  |
| 4 | The mixer Avoid dusts or splashes when mixing. |  |  |  |
| 5 | The mixer keeps containers below eye level. |  |  |  |
| 6 | He/she Never transfer pesticides into cups that may be confused with food containers. |  |  |  |
| 7 | The mixer never used additional pesticide for an increased effect |  |  |  |
| 8 | Mix the pesticide away from streams. |  |  |  |
|  | **Regarding application of spraying** |  |  |  |
| 1 | Wearing safety equipments before starting to spray: |  |  |  |
|  | Head cover |  |  |  |
|  | Goggle |  |  |  |
|  | Face shield/ respirator |  |  |  |
|  | Glove |  |  |  |
|  | Long sleeved t-shirt |  |  |  |
|  | Long pants/trouser |  |  |  |
|  | Plastic boot |  |  |  |
| 2 | The spraying containers have no spills. |  |  |  |
| 3 | Change cloths after end of spraying. |  |  |  |
| 4 | Wash skin after spraying. |  |  |  |
| 5 | Recap empty container to protect spillage. |  |  |  |
| 6 | Puncture empty container to prevent reuse for household purpose |  |  |  |
| 7 | Empty container is buried well. |  |  |  |
| 8 | Clearly label treated surfaces where residue may remain /until dried. |  |  |  |
|  | **Regarding storage of pesticides** |  |  |  |
| 1 | Storage place is dry. |  |  |  |
| 2 | Pesticide containers are tightly closed. |  |  |  |
| 3 | Pesticides are stored by its original container. |  |  |  |
| 4 | Dry pesticides are put over liquid ones. |  |  |  |
| 5 | Pesticides are place on non-absorbent shelves and upright and off the floor. |  |  |  |
| 6 | The storage house is free from flammable condition. |  |  |  |
| 7 | The storage house has locked. |  |  |  |
| 8 | Only pesticides are kept in the storage cabinet/area. |  |  |  |

Key informant interviews guide (to training facilitators)

1. Do you identify training interests of farmers regarding pesticide use safety practice?
2. If say yes, what was the interests of farmers towards pesticides use safety practice?
3. If No, why?
4. Do you give training to farmers about pesticide use safety practice?
5. If yes, what was their response next to the training?
6. If no, why?

Key informant interviews guide (to pesticide distributors)

1. Do you apply safety practice when distributing pesticides to farmers?
2. Do you tell them how to store pesticides?
3. Do you inform well the way how to mix and spray pesticides?
4. Do you access safety equipments to farmers when applying pesticides?

Key informant interviews guide (to Retailers)

1. Do you have told the farmers (your clients) seeing the labels on the pesticide containers?
2. Do you have accessed safety equipments for selling?
3. If yes, do all farmers bought the equipment?
4. If no, why?
5. Do you strictly advice the farmers using safety equipments when mixing and spraying?

Key informant interview guide (to model farmers)

1. Do you have taken training on pesticide use safety practice?
2. Where do you get safety equipments?
3. Do other pesticide sprayer farmers acquire such materials as they need?
4. Do you regularly use safety materials when spraying pesticides?
5. Do farmers in this woreda apply safe way of pesticide use?
6. If not, what were the problems?
7. What solutions are needed to promote safe practice of pesticide use?

Key informant interview guide (NGOs facilitators)

1. Do you have how many of farmers safely practice while spraying pesticides?
2. What was your contribution in promoting safe practice of pesticide spraying?
3. What type of problems you observe when you are working with farmers about pesticide use?
4. What kind of solutions do you think to expand safe use of pesticides during spray?
