# Supplementary material for: Pesticide use safety practices and associated factors among farmers in Fogera district wetland areas, south Gondar zone, Northwest Ethiopia
Source: PLoS One. 2023 Jan 10;18(1):e0280185. doi: 10.1371/journal.pone.0280185 (PMC9831305; doi:10.1371/journal.pone.0280185)
Supplement: Attachment — Submitted filename: PLUS One Reviews.pdf [file pone.0280185.s003.pdf]

PLOS ONE

Pesticide use safety practices and associated factors among farmers in fogera district wetland areas, south Gondar zone, Northwest Ethiopia 2021

--Manuscript Draft--

**Manuscript Number:**	PONE-D-22-18751
**Article Type:**	Research Article
**Full Title:**	Pesticide use safety practices and associated factors among farmers in fogera district wetland areas, south Gondar zone, Northwest Ethiopia 2021
**Short Title:**	Pesticide use safety practices and associated factors
**Corresponding Author:**	Genet Gedamu Kassie<br>Bahir Dar University<br>Bahir Dar, ETHIOPIA
**Keywords:**	pesticide use; safety measures; safety practice; wetland
**Abstract:**	Background<br><br>Farmers in developing countries including Ethiopia are exposed to agricultural pesticides, including pesticides which are restricted and banned in developed countries. There is scanty information on pesticide use safety practices and its associated factors among farmers in Ethiopia, particularly in the study area.<br><br>Objective<br><br>To assess pesticide use safety practices and associated factorsamong farmers in Fogera district wetland area, Amhara region, Northwest Ethiopia 2021.<br><br>Methods<br><br>A Community based cross-sectional study design that employs quantitative and qualitative methods from August 25-September 30 2021 were used. Four hundred thirty participants were included by using stratified random sampling technique. Pre-tested interview questionnaire, observational checklist and key informant guide were used to collect data. Data were entered into Epi data version 4.6 and analyzed using SPSS version 21. Bi-variable logistic regression was used to identify factors associated with the dependent variable. A p-value <0.05 was used as cut off point to declare statistically significant association between factors and outcome variable. Odds ratio and 95% CI were calculated to describe the strength of association between factors and outcome variable. Thirty five respondents from farmers and different expertise were participated to qualitative data and analyzed by using thematic analysis open-code 4.03 software.<br><br>Result<br><br>The proportion of good pesticide use safety practices in the study area was (24.4%: 95% CI: 21.4%-29.3%). Educational status (AOR: 3.19, 95% CI: 1.44-6.71), ever exposure of pesticide before (AOR: 6.85. 95% CI: 2.426-9.35), knowledge of pesticide usage (AOR: 3.40, 95%CI: 1.459-7.855), access to safety materials (AOR: 2.06, 95%CI: 1.198-3.536), and ever had training (AOR: 4.93, 95% CI: 2.88-8.59) were factors associated with good safety practice of pesticide use. Qualitatively, limited material access, lack of government attention, insufficient training opportunity, absence of media cover, weak enforcement of laws and limited guideline access barred good safety practice of pesticide use.<br><br>Conclusion<br><br>The study revealed that good safety practice was low in the study area. Being educated, ever exposed pesticide before, having good knowledge of pestic[illegible]usage, access of safety materials, and ever had training on pesticides use increased the odds of good practice of pesticide use. Insufficient training opportunity and material access,

Powered by Editorial Manager® and ProduXion Manager® from Aries Systems Corporation

	weak law enforcement, limited access of guidelines and shortage of media cover were challenges identified qualitatively.
**Order of Authors:**	Genet Gedamu Kassie
	Fisiha Alebachew
	Muluken Azage
	Muluken Chanie
**Additional Information:**	
**Question**	**Response**
**Financial Disclosure**<br><br>Enter a financial disclosure statement that describes the sources of funding for the work included in this submission. Review the submission guidelines for detailed requirements. View published research articles from *PLOS ONE* for specific examples.<br><br>This statement is required for submission and **will appear in the published article** if the submission is accepted. Please make sure it is accurate.<br><br>**Unfunded studies**<br>Enter: *The author(s) received no specific funding for this work.*<br><br>**Funded studies**<br>Enter a statement with the following details:<br>• Initials of the authors who received each award<br>• Grant numbers awarded to each author<br>• The full name of each funder<br>• URL of each funder website<br>• Did the sponsors or funders play any role in the study design, data collection and analysis, decision to publish, or preparation of the manuscript?<br>• **NO** - Include this sentence at the end of your statement: *The funders had no role in study design, data collection and analysis, decision to publish, or preparation of the manuscript.*<br>• **YES** - Specify the role(s) played.<br><br>* typeset	The author(s) received no specific funding for this work.
**Competing Interests**<br><br>Use the instructions below to enter a competing interest statement for this	The authors have declared that no competing interests exist

submission. On behalf of all authors, disclose any competing interests that could be perceived to bias this work—acknowledging all financial support and any other relevant financial or non-financial competing interests.<br><br>This statement is required for submission and **will appear in the published article** if the submission is accepted. Please make sure it is accurate and that any funding sources listed in your Funding Information later in the submission form are also declared in your Financial Disclosure statement.<br><br>View published research articles from *PLOS ONE* for specific examples.<br><br>**NO authors have competing interests**<br><br>Enter: *The authors have declared that no competing interests exist.*<br><br>**Authors with competing interests**<br><br>Enter competing interest details beginning with this statement:<br><br>*I have read the journal's policy and the authors of this manuscript have the following competing interests: [insert competing interests here]*<br><br>* typeset	
**Ethics Statement**<br><br>Enter an ethics statement for this submission. This statement is required if the study involved:<br><br>• Human participants<br>• Human specimens or tissue<br>• Vertebrate animals or cephalopods<br>• Vertebrate embryos or tissues<br>• Field research<br><br>Write "N/A" if the submission does not	Ethical clearance was obtained from the institutional ethical review board of Bahir Dar University College of Health Science and supporting letter was taken from Fogera woreda administrative and health office before the study started. Then informed verbal consent was obtained from the respondents after the necessary explanation about the purpose, benefits and risks of the study by the data collectors. The data collectors continued the data collection process after the respondents said yes i am volunteer to participate on the study. The confidentiality of participants' information was maintained by anonymous data.

Powered by Editorial Manager® and ProduXion Manager® from Aries Systems Corporation

require an ethics statement.<br><br>General guidance is provided below. Consult the submission guidelines for detailed instructions. **Make sure that all information entered here is included in the Methods section of the manuscript.**<br><br>**Format for specific study types**<br><br>**Human Subject Research (involving human participants and/or tissue)**<br>• Give the name of the institutional review board or ethics committee that approved the study<br>• Include the approval number and/or a statement indicating approval of this research<br>• Indicate the form of consent obtained (written/oral) or the reason that consent was not obtained (e.g. the data were analyzed anonymously)<br><br>**Animal Research (involving vertebrate animals, embryos or tissues)**<br>• Provide the name of the Institutional Animal Care and Use Committee (IACUC) or other relevant ethics board that reviewed the study protocol, and indicate whether they approved this research or granted a formal waiver of ethical approval<br>• Include an approval number if one was obtained<br>• If the study involved *non-human primates*, add *additional details* about animal welfare and steps taken to ameliorate suffering<br>• If anesthesia, euthanasia, or any kind of animal sacrifice is part of the study, include briefly which substances and/or methods were applied<br><br>**Field Research**<br><br>Include the following details if this study involves the collection of plant, animal, or other materials from a natural setting:<br>• Field permit number<br>• Name of the institution or relevant body that granted permission	
**Data Availability**	Yes - all data are fully available without restriction

Authors are required to make all data underlying the findings described fully available, without restriction, and from the time of publication. PLOS allows rare exceptions to address legal and ethical concerns. See the PLOS Data Policy and FAQ for detailed information.<br><br>A Data Availability Statement describing where the data can be found is required at submission. Your answers to this question constitute the Data Availability Statement and **will be published in the article**, if accepted.<br><br>**Important:** Stating 'data available on request from the author' is not sufficient. If your data are only available upon request, select 'No' for the first question and explain your exceptional situation in the text box.<br><br>Do the authors confirm that all data underlying the findings described in their manuscript are fully available without restriction?	
**Describe where the data may be found in full sentences. If you are copying our sample text, replace any instances of XXX with the appropriate details.**<br><br>• If the data are **held or will be held in a public repository**, include URLs, accession numbers or DOIs. If this information will only be available after acceptance, indicate this by ticking the box below. For example: *All XXX files are available from the XXX database (accession number(s) XXX, XXX.*).<br>• If the data are all contained **within the manuscript and/or Supporting Information files**, enter the following: *All relevant data are within the manuscript and its Supporting Information files.*<br>• If neither of these applies but you are able to provide **details of access elsewhere**, with or without limitations, please do so. For example:	All relevant data are within the manuscript and its Supporting Information files.

Powered by Editorial Manager® and ProduXion Manager® from Aries Systems Corporation

Data cannot be shared publicly because of [XXX]. Data are available from the XXX Institutional Data Access / Ethics Committee (contact via XXX) for researchers who meet the criteria for access to confidential data.<br><br>*The data underlying the results presented in the study are available from (include the name of the third party and contact information or URL).*<br>• This text is appropriate if the data are owned by a third party and authors do not have permission to share the data.<br><br>* typeset	
Additional data availability information:	

Powered by Editorial Manager® and ProduXion Manager® from Aries Systems Corporation

Manuscript Click here to access/download;Manuscript;Manuscript.docx

Pesticide use safety practices and associated factors among farmers in fogera district wetland areas, south Gondar zone, Northwest Ethiopia ~~2021~~

Short Title: Pesticide use safety practices and associated factors

Fisiha Alebachew[1], Muluken Azage[2], Genet Gedamu[2*], Muluken Chanie[3]

[1] Department of Nursing, Debre Tabor Health Sciences College, Debre Tabor, Ethiopia

[2] Department of environmental health, College of Medicine and Health Sciences, school of public health, Bahir Dar University, Bahir Dar, Ethiopia

[3] Department of health informatics, Debre Tabor Health Sciences College, Debre Tabor, Ethiopia

***Corresponding author**

Email: geni_31280@yahoo.com (GG)

Abstract

Background: Farmers in developing countries including Ethiopia are exposed to agricultural pesticides, including pesticides which are restricted and banned in developed countries. There is scanty information on pesticide use safety practices and its associated factors among farmers in Ethiopia, particularly in the study area.

Objective: To assess pesticide use safety practices and associated factorsamong farmers in Fogera district wetland area, Amhara region, Northwest Ethiopia 2021.

Methods: A Community based cross-sectional study design that employs quantitative and qualitative methods from August 25-September 30 2021 were used. Four hundred thirty participants were included by using stratified random sampling technique. Pre-tested interview questionnaire, observational checklist and key informant guide were used to collect data. Data were entered into Epi data version 4.6 and analyzed using SPSS version 21. Bi-variable logistic regression was used to identify factors associated with the dependent variable. A p-value <0.05 was used as cut off point to declare statistically significant association between factors and outcome variable. Odds ratio and 95% CI were calculated to describe the strength of association between factors and outcome variable. Thirty five respondents from farmers and different expertise were participated to qualitative data and analyzed by using thematic analysis open-code 4.03 software.

Result: The proportion of good pesticide use safety practices in the study area was (24.4%: 95% CI: 21.4%-29.3%). Educational status (AOR: 3.19, 95% CI: 1.44-6.71), ever exposure of pesticide before (AOR: 6.85. 95% CI: 2.426-9.35), knowledge of pesticide usage (AOR: 3.40, 95%CI: 1.459-7.855), access to safety materials (AOR: 2.06, 95%CI: 1.198-3.536), and ever had training (AOR: 4.93, 95% CI: 2.88-8.59) were factors associated with good safety practice of pesticide use. Qualitatively, limited material access, lack of government attention, insufficient training opportunity, absence of media cover, weak enforcement of laws and limited guideline access barred good safety practice of pesticide use.

Conclusion: The study revealed that good safety practice was low in the study area. Being educated, ever exposed pesticide before, having good knowledge of pesticide usage, [illegible]ess of safety materials, and ever had training on pesticides use increased the odds of good practice of pesticide use. Insufficient training opportunity and material access, weak law enforcement, limited access of guidelines and shortage of media cover were challenges identified qualitatively.

Key words: pesticide use, safety measures, safety practice, wetland

Introduction

Pesticides are drugs used for preventing and controlling pests, weeds, vectors, rodents, insects in agriculture to increase productivity and also applied in the household(mosquitoes, ticks, rats, lice) to kill them (1). ~~Pesticide~~ Their use safety practice justifies all procedures, actions, policies applied to minimize the risk of exposure to potentially hazardous pesticides (2). Pesticide use safety practice can be also illustrated by application of appropriate personal hygiene, effective laundry, separate pesticides storage home , using advisable concentration and quantity based on labeling, avoid eating and drinking at the time of spray, proper use of personal protective equipment(PPE), appropriate disposal of empty containers (3). Worldwide, it is estimated that approximately 1.8 billion people engage in agriculture and most use pesticides to increases their productivity (4). ~~Per year 5.6 billion pound of pesticides utilized globally for herbicides (weeds), insecticides (insects), fungicides (fungi), and microbicides (5)~~.

During the last two decades, international bodies have taken up the issues and adopted a number of solutions and programs to address the effects of pesticide use. Despite these efforts, global pesticide use has continued to grow steadily to 4.1 million tons per year in 2017(6).Across the world, the status of farmers pesticide use safety practices were insufficient: 43.1% in Nepal 42%Kuwait, 50.8% in Iran, 61% in Uganda and Costa Rica, and 26.6 % in Ethiopia.(13-16).Following this, the global impact of inappropriate handling of pesticide was estimated to be amount of 155,488 deaths and 7,362,493 disability-adjusted life years (DALYs) in 2016 (17).

Farmers in developing countries still continue to use pesticides at increasing quantities because of ignorance of sustainability of pesticide use, lack of alternatives to pesticides, underestimation of short and long-term effects of pesticide use and weak enforcement of laws and regulations(6).Regarding African countries, importing interests of pesticides is highly escalating. To the reverse, the program of controlling pesticides is limited. The reason behind this is that users have no information about the purpose of each pesticide product, hazard level(toxicology class), dosage and concentration, way of protection, access to protective equipment(9, 10).

In Ethiopia, there is no integrated poisoning center of reporting system and disease hampering estimation institutions particularly on pesticides effect on health and the environment of the community. This is a clear indication of giving less attention to pesticide-related health impacts

and insignificant intervention towards safe pesticide use practice in agriculture(11, 12). Studies reported that farmers having favorable attitude on pesticide use safety practice had good precaution, used safety equipment and safely used pesticides compared to those who had unfavorable ones(10, 12, 37, 40)

Whatever efforts have been undertaken, pesticide users of Ethiopians in general and the study area of Fogera wetland in particular [illegible]avily exposed to short-term (e.g., skin and eye irritation, headaches, dizziness, and nausea) and long-term(e.g., cancer, asthma, and diabetes) pesticide effects (3). Furthermore, farmers in wet land areas of fogera district grow different products two to three times a year and they have intensively and frequently utilized pesticides for their productivity of potatoes, onions, cabbages, different vegetables, and chat growth but there is scarcity information on pesticide use safety practices. Moreover, fewer studies conducted in Ethiopia and more focused on flower farm/commercial farm workers of pesticide use. (26). Therefore, the aim of the study was to assess pesticide use safety practices and its associated factors, as well as explore challenges of pesticide safety practice in Fogera wetland area.

Methods and Materials

Study setting and period

The study was ~~employed on~~ done in Fogera Woreda wetland from August25-Setember 30/ 2021, South Gondar, Northwest Ethiopia.Fogera Woreda lay to the south-eastern shore of Lake Tana on the road from Bahir Dar to Gondar, 625 km from Addis Ababa, and 55 km north of the Regional capital of Bahir Dar city. The district is bordered by LibokemkemWoreda in the north, Dera Woreda in the south, Lake Tana in the west and Farta Woreda in the east. Location Map of Ribb-Gumara rivers of Fogera Woreda lay to the south-eastern shore of Lake Tana. The well-known towns in Fogera Woreda are Woreta and Alem Ber, the former being the headquarter of the Woreda. Such district has thirty three Peasant Associations (PAs) or Kebeles and city administration having five kebeles. The area is located between 11° 57' N and 12 0 30' N latitude and 37° 35' E and 37 0 58' E longitude.

25th August to 30th September 2021

Study design and population

A community-based cross-sectional study triangulated by qualitative study was employed in fogera Woreda wetland farmers. All farmers who were using and applying the pesticides for agricultural practices on their ~~agricultural~~ farmlands at least in the last one year from period of

data collection were included. Whereas farmers unable to communicate due to illness were excluded in the study.

Sample size determination and sampling procedure

For the quantitative data, ,the required sample size was calculated using a single population proportion formula(n)= $(Z\ a/2)^2\ P(1 - P)/d^2$ via the following assumptions: The proportion from the pretested result done on shagakebele in Fogera district 21.7%, 95% confidence level, 5% margin of error, and 10% estimated non–response rate (16). Considering a design effect of 1.5, the calculated sample of farmers was 430.

For the qualitative part, purposively 35(30 males and 5 females) participants were selected from W woreda and K kebele training facilitators (6), 6 K kebele pesticides distribution center officials (6), private pesticide retailers in the town (5), model farmers using safety equipment (10), leaders of farmer associations (5),NGO facilitators (3).

Data collection tools and procedure

Quantitative data were collected by using a pre-tested semi-structured questioner derived from previous literatures (11, 12, 16, 20, 41)through face-to-face interviews at home. The questionnaire was design in English but the interviews were conducted in the local language, (Amharic) and back to English for consistency of data analysis. Data were collected by 5 trained diploma Agricultural sector workers and supervised by 1 trained BSC Environmental Health Professional within the period of the research. from August 25 –September 30/2021. Whereas the qualitative data was collected by using in-depth interview and key informants interview guide related to farmers' pesticide use safety practices. Participants were asked particularly on exploring barriers on farmers' pesticide use safety practices. A standard observation checklist was also implemented to ensure pesticide use safety implementation of farmers when mixing, spraying, and disposal of empty containers.

Data quality assurance

Data quality was assured using data collectors training about the overall process of data collection. The questionnaire was first prepared in English language and then translated to 'Amharic' language, which was the local language of the study, subjects, and back to English by language experts to check their consistency and conceptual equivalence. Amharic version of the questionnaires was used to obtain data from participants. The data collectors were supported by supervisors and prompt feedback. Each completed questionnaire was cheeked for coherence, completeness, consistency at the same time. The daily evaluation was performed to correct any problem that

could face during the course of data collection and the pretest was conducted 5% of the population of shaga kebele, which was not selected as a study population within the study areas.

Operational definitions

Pesticides use safety practice: is protecting and minimizing exposures of pesticides' effect when applying it on agriculture at least by using hat, goggle, facemask, long-sleeved shirts and trousers, gloves, boots; separate storage of pesticides and properly disposed of empty pesticide containers (12).

Data processing and analysis

Quantitative data were coded and entered into Epi-data version 4.6 statistical software. It is cleaned, edited and analyzed by using SPSS Version 21 statistical software. To explain the study population with relevant variables, descriptive statistics were used. All variables with a p-value <0.25 in the bivariable logistic regression analysis were used for multivariable analysis in order to increase the number of independent variables that was associated with dependent variable. P-values of less than 0.05 were considered statistically significant. Multivariable binary logistic regression analyses and adjusted odds ratios with corresponding 95% confidence level (CL) were used to determine and report strength of association between dependent and independent variables. For qualitative part thematic method and open code-4.03, software was used. Data in the form of audio-files/field note obtained from the participants was transcribed to the Amharic language (transcribed word by word), and then translated into English language. Before analyzing the data, first, all the contents of the transcripts were read repeatedly. Then coding was performed. After coding and displaying the data to get the overall sense, data reduction was done to make the most essential concepts and relationships. Finally, interpretation was performed.

Ethical considerations

Ethical clearance was obtained from the institutional ethical review board of Bahir Dar University College of Health Science and supporting letter was taken from Fogera woreda administrative and health office before the study started. Then informed verbal consent was obtained from the respondents after the necessary explanation about the purpose, benefits and risks of the study by the data collectors. The data collectors continued the data collection process after the respondents said 'yes I i am volunteer to participate on the study'. The confidentiality of participants' information was maintained by anonymous data.

Results

Socio-demographic characteristics

The response rate of this study was 430 (100%). Four hundred eighteen (97.2%) respondents were males and 344(80%) were rural residents. Three hundred twenty seven (76%) of the respondents were Orthodox Christians. Among all participants, One hundred sixty (37.2%) ~~were~~ can not read and write, and 325(75.6%) were married (**Table1**).

Table 1: socio-demographic characteristics respondents in Fogera district wetland areas, Northwest Ethiopia, 2021(n=430)

Variables	Categories	Frequency (Percentage)
**Residence**	Urban	86(20%)
	Rural	344(80%)
**Sex**	Male	418(97.2)
	Female	12(2.8%)
**Age in Years**	18-30	173(40.3%)
	31-40	191(44.4%)
	41-50	59(13.7%)
	>50	7(1.6%)
**Marital Status**	Single	57(13.3%)
	Married	325(75.6%)
	Divorced	36(8.4%)
	Widowed	12(2.8%)
**Religion**	Orthodox	327(76%)
	Muslim	77(17.9%)
	Catholic	8(1.9%)
	Protestant	9(2.1%)
	Other(Adventist)	9(2.1%)
**Educational Status**	Can’t read and Write	160(37.2%)
	Can read and write	78(18.1%)
	Primary Education	71(16.5%)
	Secondary Education	61(14.2%)
	Diploma and Above	60(14%)

**Experience of pesticide Spray**	< 6years	156(36.3%)
	6-10Years	141(32.8%)
	>10 Years	133(29.9%)
**Income in months**	1500-2000	120(27.9%)
	2001-3000	101(23.5%)
	>3000	209(48.6%)
**Working hours of Spray in a day**	1-4Hours	128(29.8%)
	5-8Hours	188(43.7%)
	>8Hours	114(26.5%)
**Farm size of spray**	<One hectare	96(22.3%)
	One hectare	90(20.9%)
	>One hectare	244(56.8%)
**Trend of using pesticides**	Increasing	325(75.6%)
	No change	105(24.4%)

Knowledge of respondents on pesticide use safety practice

Out of 430 respondents, 315(73.3%) had adequate knowledge about safe pesticides use practice and those who had taken training about safe practice of pesticide use were 171(39.8%). Among respondents, 238(55.3%) of them could read pesticide labels on the containers. One hundred ninety two (44.7%) of participants had knowledge about prohibited pesticides and 144(33.5%) responded about having knowledge on guidelines of safety applications. Two hundred sixty seven (62.1%) of involved recruits identified the route of entrance of pesticides into their body and 149(34.7%) of them knew safety measures on pesticide use **(Table2).**

Table 2: ~~k~~Knowledge based factors on pesticides use safety practice in Fogera district farmers of wetland area, Northwest Ethiopia ~~2021~~.

Variables	Categories	Frequency(Percent)
**Know[illegible]mes of pesticides**	No	61(14.2%)
	Yes	369(85.8)
**Know pesticides affect human health**	No	105(24.4)
	Yes	325(75.6%)
**Know pesticides affect environments(water bodies)**	No	124(28.8%)

	Yes	306(71.2%)
**Read pesticide labels on container**	No	127(44.7%)
	Yes	238(55.3%)
**Know guidelines of safety applications on pesticide use**	No	286(66.5%)
	Yes	144(33.5%)
**Know rout of pesticides enter your body**	No	163(37.9%)
	Yes	267(62.1%)
**Know recommended dose of pesticides on labels**	No	268(62.3%)
	Yes	162(37.7%)
**Know safety measures of using pesticides**	No	281(65.3%)
	Yes	149(34.7%)
**Know wearing protective equipments while mixing and spraying**	No	60(14%)
	Yes	370(86%)
**Know changing cloths after spraying pesticide**	No	105(24.4%)
	Yes	325(75.6%)
**Know washing hands after spraying pesticide**	No	45(10.5%)
	Yes	395(89.5%)
**Know take shower next to pesticide spraying**	No	150(34.9%)
	Yes	280(65.1%)
**Know types of prohibited pesticides**	No	238(55.3%)
	Yes	192(44.7%)
**Take training on safe pesticide usage**	No	259(60.2%)
	Yes	171(39.8%)
**can identify sources of information about safety practice of pesticide use**	No	279(64.9%)
	Yes	151(35.1%)
**Overall knowledge**	Adequate	315(73.3%)
	Inadequate	115(26.7%)

Attitude of respondents on pesticide use safety practice

Of the total 430 participants, 353(82.1%) of them had a favorable attitude on using pesticides safely. Two hundred eighty six (53.2%) participants wanted to buy safety equipment when

accessible. Respondents interested to wear protective equipment were 352(81.9%). Besides, 289(67.2%) of them desired to wash hands after spraying (**Table3**).

Table 3: ~~a~~ttitudes of farmers on pesticides use safety practice in ~~f~~ogera district wetland areas, Northwest Ethiopia ~~2021~~.

Variables	Categories	Frequency(Percentage)
**[illegible]ar pesticides affect your health**	Strongly disagree	55(12.8%)
	Disagree	90(20.4%)
	I don't know	3(0.8%)
	Agree	265(60.8%)
	Strongly agree	22(5.2%)
**Give attention to informations written on containers**	Strongly disagree	51(11.9%)
	Disagree	99(23.2%)
	I don't know	5(1%)
	Agree	241(56%)
	Strongly agree	34(17.9%)
**Interested to wear protective equipments**	Strongly disagree	20(4.7%)
	Disagree	55(12.8%)
	I don't know	3(0.7%)
	Agree	310(72.1%)
	Strongly agree	42(9.8%)
**Have positive feeling towards instructions about safety pesticide handling**	Strongly disagree	16(3.7%)
	Disagree	75(15.2%)
	I don't know	9(2.09%)
	Agree	294(86.4%)
	Strongly agree	46(10.7%)
**Interested in sharing information to safely handle pesticides**	Strongly disagree	39(9.1%)
	Disagree	120(27.9%)
	I don't know	2(0.5%)
	Agree	227(52.8%)
	Strongly agree	42(9.8%)

**Perceiving that safe use of pesticides protect the environment**	Strongly disagree	16(3.7%)
	Disagree	70(16.3%)
	I don't know	4(0.9%)
	Agree	241(56%)
	Strongly agree	178(41.4%)
**Interested to buy safety equipments**	Strongly disagree	77(17.9%)
	Disagree	102(24.8%)
	I don't know	7(1.6%)
	Agree	195(45.4%)
	Strongly agree	49(11.4%)
**Interested to change cloths after you have used during spraying**	Strongly disagree	38(9.9%)
	Disagree	99(12.3%)
	I don't know	4(0.9%)
	Agree	266(61.9%)
	Strongly agree	23(5.3%)
**Interested to wash hands after pesticide spraying**	Strongly disagree	8(1.9%)
	Disagree	29(6.7%)
	I don't know	2(0.5%)
	Agree	326(75.8%)
	Strongly agree	65(15.1%)
**Interested to take shower after spraying**	Strongly disagree	20(4.7%)
	Disagree	94(21.8%)
	I don't know	5(1.2%)
	Agree	260(60.5%)
	Strongly agree	51(11.9%)
**Overall attitude**	Favorable	353(82.1%)
	Unfavorable	77(17.9%)

Environmental related variables

Three hundred (69.8%) of participants gave care for weather condition while spraying and 66(15.3%) of them store pesticides in a separate dry place and closed room reach out of children. 61(14.1%) respondents bury empty containers in the ground properly(**Table4**).

Table 4: environmental factors on safety practices of pesticides use in fogera district among farmers of wetland areas, Northwest Ethiopia 2021.

Variables	Categories	Frequency(Percent)
**Care of weather condition while spraying**	No	130(30.2%)
	Yes	300(69.8%)
**Place of storing pesticides**	Bed room	63(14.7%)
	Living room	74(17.2%)
	Kitchen room	145(33.7%)
	Separate room	66(15.5%)
	Other	82(19.1%)
**Duration of storage of pesticides**	6months	227(52.8%)
	6-12months	110(25.6%)
	12-24months	77(17.9%)
	Unlimited time	16(3.7%)
**Disposing empty containers**	No	187(43.5%)
	Yes	243(56.5%)
**If yes, how do you disposing empty containers**	Burning	27(6.3%)
	Burying	61(14.2%)
	Leave on farm area	147(34.2%)

Safe practices of using pesticides

Out of 430 farmers taking part in the study, 105(24.4%: 95% CI: 21.4%-29.3%) had good safety practice in using pesticide. Among all study subjects, 109(25.3%) had regularly used personal protective equipment and 108(25.2%) of them applied safety instructions while spraying pesticide. One hundred eighty seven (43.5%) respondents answered change their cloths after spraying and 175(40.7%) of them take shower following spray pesticides. Two hundred seventy (62.8%) and 119(27.7%) participants had mixed pesticides on farm areas and using sticks by wearing glove respectively (**Table-5**).

Table 5: practice related questions on pesticide use among farmers in Fogera district wetland areas, Northwest Ethiopia 2021.

Variables	Categories	Frequency (Percentage)
	No	254(59.1%)

Always used measuring tool to add the exact amount of pesticide mentioned on the label	Yes	176(40.9%)
Place of mixing pesticides for spraying	Near water source	100(23.3%)
	On farm areas	270(62.8%)
	In the house	60(14%)
Ways of mixing pesticides	With a stick but bare hands	234(54.4%)
	With bare hands	32(7.4)%
	With hands by wearing glove	45(10.5%)
	With stick by wearing glove	119(27.7%)
Type of device used for mixing pesticides	Knapsack	362(84.2%)
	Bucket	68(15.8%)
Regularly used protective equipment while spraying	No	321(74.7%)
	Yes	109(25.3%)
Applied safety instructions on pesticides use	No	322(74.7%)
	Yes	108(25.1%)
Follow safety procedures while spraying	No	320(74.7%)
	Yes	110(25.3%)
Check safety equipment's wellness before use	No	315(73.3%)
	Yes	115(26.7%)
Change wearing cloths after spraying pesticides	No	243(56.5%)
	Yes	187(43.5%)
Wash hands after spraying	No	255(59.3%)
	Yes	175(40.7%)
When do you take shower after spraying pesticides	Sometimes	122(28.4%)
	Always	57(13.3%)
Pesticide use Safety practices score	Poor practice	325(75.6%)
	Good practice	105(24.4%)

Factors associated with safety practices on pesticide use

In the bivariable logistic regression; age, educational status, ever exposed pesticide before(spraying experience), income, length of time of spraying, farm size, ever had training on pesticide use, weather conditions, storage place of pesticides, accessibility of protective equipment, knowledge and attitude on safety practice have a p-value of <0.25. Those were candidate to multiple binary logistic regression. Out of these candidates computed with multivariable binary logistic regression, educational status, spraying experience, training on pesticide use, and accessibility of protective equipment and knowledge of using pesticides had association with safety practices of pesticide use. The odds of pesticide use safety practices among diploma and above (AOR=3.19, 95%CI: 1.44-6.71) were 3.19 times more safely practice as compared with farmers who can't read and write. Farmers who had ever exposed pesticide more than 10years (AOR=5.2, 95%CI: 2.43-9.35) were 5.2 times more likely to safely using pesticides than 5years experience. The odds of safe practices of respondents ever had training on pesticide use (AOR=4.98, 95%CI: 2.88-8.59) were 4.98 times more using pesticide in safe way as compared with farmers no training. Farmers who had accessibility of protective equipment in using pesticide (AOR=2.06 95% CI: 1.20-3.54) were 2.06 times safely practice than who had not accessed. Participants who had adequate knowledge about safety practices of pesticide use (AOR=3.40, 95% CI: 1.46-7.86) were 3.40 times more used pesticides safely compared with poor knowledge (**Table 6**).

**Variables**	**Response categories**	**Safety practices of pesticide use(n=430)**		**COR(95% CI)**	**AOR(95% CI)**
		Poor	Good		
Educational status	Can't read and write	135	25	1	1
	Informal education	71	7	0.532(.22-1.291)	0.416(0.158-1.094)
	Primary education(1-8)	44	27	3.314(1.744-6.295)	3.166(1.494-6.71)*
	Secondary education(9-12)	37	24	3.0503(1.796-6.83)	3.129(1.423-6.882)*
	Diploma and above	38	22	3.126(1.589-6.15)	3.187(1.443-7.036)*

Spray experience	6month-5years	138	18	1	1
	6-10years	103	38	2.828(1.527-5.238)	2.351(1.151-4.8)*
	>10years	84	49	4.6(1.598-6.86)	5.188(2.004-13.431)**
Training	No	228	31	1	1
	Yes	97	74	5.611(3.465-9.085)	4.975(2.88-8.593)**
Access of PPE	No	226	48	1	1
	Yes	99	57	2.711(1.727-4.255)	2.058(1.198-3.536)*
Knowledge	Poor	107	8	1	1
	Good	218	97	5.951(2.791-12.68)	3.397(1.469-7.855)*

Table 6: Factors associated with pesticide use safety practice showing crude odds ratio and adjusted odds ratio, Fogera district Northwest Ethiopia 2021.

Key: *=siginificant with p-value <0.05, ** significant with p-value<0.001, 1=reference.

Qualitative finding of safety pesticide use practice

Two central themes were created that describe safety practice of pesticide use explored by respondents: Reasons inhibit the use of safety equipment and Methods promoting safety practice of pesticide use. Subthemes under each categories; reasons inhibit the use of safety equipment(subthemes: less attention of mass medias, weak law enforcement, limited access of guidelines, insufficient availability of safety equipment, limited training opportunity, low level of understanding about long term effect of pesticides, unacceptability of safety equipment, absence of role model, uncomfortable to use), methods promoting safety practice of pesticide use(subthemes: access of safety equipment, training opportunity, attitude change, information sharing, encouraging model safety equipment users) (**Table7**).

Table 7: The outcome space showing the ordered internal relationship between the three emerging categories of description in Fogera district wet land, Northwest Ethiopia 2021.

Experience of residents about pesticide use safety measures	
subthemes/coding	Themes

less attention of mass medias<br>weak law enforcement<br>limited access of guidelines<br>insufficient availability of safety equipments<br>limited training opportunity<br>low level of understanding about long term effect of pesticides<br>unacceptability of safety equipments<br>absence of role model<br>uncomfortable to use	Reasons inhibit the use of safety equipments
access of safety equipment's<br>strong law enforcement<br>training opportunity<br>attitude change<br>information sharing<br>encouraging model safety equipment users	Methods promoting safety equipment's use

Theme 1: Reasons inhibiting safety practice of pesticide use

The problem of using safety equipment while spraying pesticides were plenty of in type. One of the problem issued by respondents were limited access of safety materials. A 40years old male farmers' association leader (participant2) noted that:

"*The woreda agricultural office given training on how to use safety equipments by showing demonstration. But they do not access safety materials to pesticide users.*"

Another farmers' association leader (participant 3) confirmed the limited access of safety equipments in such a way:

*"As a solution, our farmers' association union brought safety equipments to pesticide users but still not adequate. Many farmers used their own traditional alternatives like '**fota'** as hat and face mask, '**Guant**' as glove, their usual cloths of trouser and long-sleeved shirt as protective means."*

A 28 years old female model farmer (participant 8) described that:

"I have been using safety equipments that have given from Moonlight economic development association (MEDA) training center. But most farmers tried to protect themselves by their own experience of following wind direction and conducive time to spray."

A 25years old female pesticide retailer (participants 7) explained: *"I do not access safety equipments. Because, my clients did not asked me bringing it."*

A 35 years old female model farmer (participant 5) addressed that: *"The woreda agricultural office and some NGOs trained us the effect of pesticides and ought to use safety equipments when spray pesticides. But they do not access protective equipments at adequate level."*

A 31 years old male kebele training facilitator (participant 1) justified that: *"Certain number of farmers had interested of using safety measures since they had seen the effect but budget constraint of the woreda taken as greatest problem that handicapped protective equipment access."*

Training constraints about safety measures to all pesticide sprayers are repeatedly raised by many respondents. A 28 years old male kebele training facilitator (participant 3) stated that: *"The woreda agricultural office in conjunction with some NGOs provided training on pesticide use safety practice but still many farmers had not got any training.*"

Participants also justified that ignored law enforcement about pesticide use safety practice is another restrictive factor. A 35 years old male MEDA training facilitator commented that: *"In my view, one of farmers' exposures to pesticide effects is weak enforcement of law and lack of mass media attention towards its effect. No one obliged pesticide sprayers to apply it. They simply spray based on their experience."*

A 38 years old male model farmer (participant 6) explained: *"In my imagination, not only poor law enforcement but also absence of guidelines how to apply pesticides, exacerbated level of exposure on pesticide sprayers."*

A 32 years old male kebele pesticide distributor (participant 2) mentioned that: "*No one indoctrinated pesticide sprayers in using safety materials. Even there is no any established system in Fogera district to enforce them practicing safety measures."*

A 30 years old male model farmer responded that: *"many farmers spraying pesticides had not accepted the use of safety equipment due to suffocation discomfort."*

Theme 2: Methods promoting safety pesticide use practice

Law enforcement and working on behavioral change empowered safety practice. **A** 32 years old male woreda training facilitator (participant1) mentioned that: "*I believe that pesticide use safety practice can be implemented when there is strong law enforcement and doing more on attitude change towards sprayers".*

A 28 years old male woreda pesticides distributor (participant 2) explained that*: "Until behavioral change comes among pesticide sprayers, strong obligatory law enforcement is needed."*

A 35 years old male organization of rehabilitation and development Amhara (ORDA) facilitator (participant 3) stated that*: "The number of farmers using safety equipment while pesticide spraying might increase when concerned government structure working with NGOs doing on pesticide protection."*

Participants also commented that the district government offices should allocate budget for pesticide protective material supply/access.

A 25 years old male model farmer in using safety materials (participant4) remark that*: "From the time that MEDA organization gave me safety equipment, I regularly apply safety measures and many pesticide sprayers had greatest interest to use if they got access."*

A 32 years old male model farmer (participant 6) explained that*: "In the beginning, safety materials had not comfortable to use. But now I adopted it and do not spray pesticides without using it. By observing me, other farmers inspired to use safety equipment's as they have got the chance."*

A 28 years old male model farmer (participant 3) expressed that*: "Farmers in fogera district have no problem of income to buy safety equipments. Hence, the concerned body tried to change attitude of farmers and provide access of materials to them."*

Respondents also underlined the imperatives of training that enable to advance awareness and attitude of pesticide sprayers.

A 28 years old male kebele training facilitator (participant 2) remarked that: *"In addition to lesson learned from experience, training empowers farmers' inspiration to use safety equipment while spraying pesticides."*

A 35 years old male model farmer (participant 6) mentioned that: *"After training, I have applied complete safety equipment including cover all. Even I have shared ideas to my neighbors how much conducive to be free from any pesticide symptoms."*

Discussion

This study tried to assess the prevalence and associated factors of pesticide use safety practices among farmers inFogera district wet land areas and also tried to explore challenges of safe pesticide use practices in the study area.This study revealed that the prevalence of safety

practice was 24.4%(95%,CI: 21.4%-29.3%). Educational status, spraying experience, ever had training on pesticide use, accessibility of protective equipment, knowledge of using pesticides are associated with pesticide use safety practice. The qualitative study also reported that equipment access taken as crucial issue for safety practices of pesticide use. This study was consistent with the study done in south-west showa and East showa, Ethiopia (26.6%),(28.1%) respectively (16, 42).

The finding of such study was higher than research done Northwest Ethiopia(8.29%) and rice farmers in Iran (8.6%) (20, 43). This disparity might be the time gap of studies done and difference of study subjects involved in the studies.But this study was less than study done in Bahirdar city and Gondar city, Ethiopia (61.3%) (63.8) respectively (36, 44). Such discrepancy might be due to study subject difference, organizational access of safety equipments as well as having good access of training since the studies done in flower farm workers.

The result of safety pesticide use practices of this study were also lower than study done in Uganda (55%), Costarica (61%), Iran (50.8%), Nepal (43.1%), and Kuwait (42%) (13-15, 20). This disparity might be due to research setting, educational level of study individuals, Economic and socio-demographic difference.

In this study, educational status of diploma and above is positively associated with pesticide use safety practices. Pesticide sprayers having diploma and above have more safely practice than uneducated farmers. This study was supported by south west showa, Ethiopia, Nepal, Nigeria (13, 15, 16). The reason for this might be more educated farmers have prior knowledge about the toxic effects of pesticides through formal education than uneducated farmers. In addition, educated farmers can easily accept changes, trainings given and practiced well than uneducated ones.

Spraying experience of farmers had also significantly associated with safety practice of pesticide use. Farmers spraying more than 10 years had more safely practice pesticide spraying than 5 years’ experience. It was supported by study done in Cameroon and Iran (5, 15). Similarly, it was supported by qualitative observation data. The justification behind this could be farmers having longer years of pesticide spraying exposure would clearly see effects of unsafe pesticide use result. Furthermore, they would get more information about the importance of safety practice of pesticide use from different sources through these times and they could

develop greater interest of saving themselves being vulnerable to pesticide residuals and tried to protect themselves from such bad consequence.

Ever had training and good knowledge towards pesticide usage were statistically significant with pesticide use safety practice. Farmers took training on safe way of pesticide application were more safely practice than who did not take. It also recognized by qualitative observational data. Such conditions were supported by study done in Nepal (13). The reason might be farmers taking training on how to safely use pesticides increase their awareness and build good knowledge to spray it as well as practice better. In such study, accessibility of safety equipment was positively associated with pesticide use safety practice. This was supported by qualitative data. It was supported by study done in southwest Showa, Ethiopia, Uganda (14, 16). Whatever pesticide sprayers had good knowledge and attitude to safety practice and protect themselves from pesticides, without accessibility and availability of safety materials everything is dream. As revealed from the qualitative study, farmers who used pesticides had acquired safety equipment from government, NGOs, private retailers and farmers' association distribution centers but still they did not satisfied by accessibility of safety equipment to protect themselves from pesticide effects.

Conclusion

The study revealed that good safety practice was low in the study area. Being educated, ever exposed pesticide before, having good knowledge of pesticide usage, access of safety materials, and ever had training on pesticides use increased the odds of good practice of pesticide use. Insufficient training opportunity and material access, weak law enforcement, limited access of guidelines and shortage of media cover were challenges identified qualitatively. These identified modifiable factors are the area of intervention to strengthen and device intervention to increase good pesticide use safety practice.

Acknowledgments

The authors acknowledged Bahir Dar University, College of Medicine and Health Sciences, School of public health for support and facilitating of this study. The authors also acknowledged data collectors, supervisor and study participants for their contribution of this work.

Availability of data and materials

As a basic data set, the data used to create this publication will be attached upon request.

Authors' Contributions

FY, worked on the conception of the research idea, designing the study, involved in proposal writing, training and supervising the data collectors, analyzed and interpreting the results. MA, MC and GG played their role in critically revising the proposal, participated in its design, analyzed and interpreting the results and wrote the manuscript. All authors were involved in reading and approving the final manuscript.

Corresponding author

Genet Gedamu

School of Public Health, College of Medicine and Health Sciences, Bahir Dar University

Email: geni_31280@yahoo.com

Consent for publication

Not applicable

Declaration/competing interest

The authors declare that they do not have any conflict of interest in any aspect of the article

Abbreviations and acronyms

AOR: Adjusted Odds Ratio,CI: Confidence Interval ,CL: Confidence Level,DALY: Disability-Adjusted Life Years,MEDA: Moonlight Economic Development Association,NGO: Non-Governmental Organization,SPSS: Statistical Package for Social Study,

Reference

1. Negatu B, Vermeulen R, Mekonnen Y, Kromhout H. Neurobehavioural symptoms and acute pesticide poisoning: a cross-sectional study among male pesticide applicators selected from three commercial farming systems in Ethiopia. Occupational and environmental medicine. 2018;75(4):283-9.
2. Smith KR. Health, safety, and environmental legislation in the UK and Europe. JCT: Journal of coatings technology. 1990;62(788):77-81.
3. Gesesew HA, Woldemichael K, Massa D, Mwanri L. Farmers knowledge, attitudes, practices and health problems associated with pesticide use in rural irrigation villages, Southwest Ethiopia. PloS one. 2016;11(9):e0162527.
4. Damalas CA, Koutroubas SD. Farmers' training on pesticide use is associated with elevated safety behavior. Toxics. 2017;5(3):19.

5. Oluwole O, Cheke RA. Health and environmental impacts of pesticide use practices: a case study of farmers in Ekiti State, Nigeria. International journal of agricultural sustainability. 2009;7(3):153-63.
6. Atreya K. Pesticide use knowledge and practices: A gender differences in Nepal. Environmental Research. 2007;104(2):305-11.
7. Deknock A, De Troyer N, Houbraken M, Dominguez-Granda L, Nolivos I, Van Echelpoel W, et al. Distribution of agricultural pesticides in the freshwater environment of the Guayas river basin (Ecuador). Science of the Total Environment. 2019;646:996-1008.
8. Houbraken M, Bauweraerts I, Fevery D, Van Labeke M-C, Spanoghe P. Pesticide knowledge and practice among horticultural workers in the Lâm Đồng region, Vietnam: A case study of chrysanthemum and strawberries. Science of the Total Environment. 2016;550:1001-9.
9. Mengistie BT, Mol AP, Oosterveer P. Pesticide use practices among smallholder vegetable farmers in Ethiopian Central Rift Valley. Environment, Development and Sustainability. 2017;19(1):301-24.
10. Memon QUA, Wagan SA, Chunyu D, Shuangxi X, Jingdong L, Damalas CA. Health problems from pesticide exposure and personal protective measures among women cotton workers in southern Pakistan. Science of the Total Environment. 2019;685:659-66.
11. Rijal JP, Regmi R, Ghimire R, Puri KD, Gyawaly S, Poudel S. Farmers' knowledge on pesticide safety and pest management practices: A case study of vegetable growers in Chitwan, Nepal. Agriculture. 2018;8(1):16.
12. Staudacher P, Fuhrimann S, Farnham A, Mora AM, Atuhaire A, Niwagaba C, et al. Comparative Analysis of Pesticide Use Determinants Among Smallholder Farmers From Costa Rica and Uganda. Environmental Health Insights. 2020;14:1178630220972417.
13. Taghdisi MH, Besheli BA, Dehdari T, Khalili F. Knowledge and practices of safe use of pesticides among a group of farmers in northern Iran. The international journal of occupational and environmental medicine. 2019;10(2):66.
14. Geleta DH, Alemayehu M, Asrade G, Mekonnen TH. Low levels of knowledge and practice of occupational hazards among flower farm workers in southwest Shewa zone, Ethiopia: a cross-sectional analysis. BMC public health. 2021;21(1):1-12.
15. Boedeker W, Watts M, Clausing P, Marquez E. The global distribution of acute unintentional pesticide poisoning: estimations based on a systematic review. BMC public health. 2020;20(1):1-19.
16. Sharifzadeh MS, Abdollahzadeh G, Damalas CA, Rezaei R, Ahmadyousefi M. Determinants of pesticide safety behavior among Iranian rice farmers. Science of the Total Environment. 2019;651:2953-60.
17. Abang A, Kouame C, Abang M, Hannah R, Fotso A. Vegetable growers perception of pesticide use practices, cost, and health effects in the tropical region of Cameroon. International Journal of Agronomy and Plant Production. 2013;4(5):873-83.
18. Mequanint C, Getachew B, Mindaye Y, Amare DE, Guadu T, Dagne H. Practice towards pesticide handling, storage and its associated factors among farmers working in irrigations in Gondar town, Ethiopia, 2019. BMC research notes. 2019;12(1):1-6.
19. Ali MP, Kabir MMM, Haque SS, Qin X, Nasrin S, Landis D, et al. Farmer's behavior in pesticide use: Insights study from smallholder and intensive agricultural farms in Bangladesh. Science of the Total Environment. 2020;747:141160.
20. Shajua M, Laohasiriwong W. Patterns of Chemical Pesticide Use and Determinants of the Use of Personal Protective Equipment to Minimize Chemical Exposure in Vegetable Farming, Maldives. Medico-Legal Update. 2021;21(2).

21. Damte T, Tabor G. Small-scale vegetable producers' perception of pests and pesticide uses in East Shewa zone, Ethiopia. International Journal of Pest Management. 2015;61(3):212-9.
22. Agmas B, Adugna M. Attitudes and practices of farmers with regard to pesticide use in NorthWest Ethiopia. Cogent Environmental Science. 2020;6(1):1791462.
23. Endalew M, Gebrehiwot M, Dessie A. Pesticide Use Knowledge, Attitude, Practices and Practices Associated Factors Among Floriculture Workers in Bahirdar City, North West, Ethiopia, 2020. Environmental Health Insights. 2022;16:11786302221076250.

Click here to access/download
Supporting Information
S1 Minimal Data Set.sav
